# Chronic Otitis Media Resulting in Aortic Valve Replacement: A Case Report

**Published:** 2015-04-03

**Authors:** Adem Guler, Mehmet Ali Sahin, Fahri Gurkan Yesil, Uzeyir Yildizoglu, Sait Demirkol, Mehmet Arslan

**Affiliations:** *Department of Cardiovascular Surgery, Gulhane School of Medicine, Ankara, Turkey.*

**Keywords:** Bicuspid aortic valve, Otitis media, Chronic disease, Carnobacteriaceae, Endocarditis, bacterial

## Abstract

The bicuspid aortic valve is known to be the most common congenital cardiac malformation, with an approximate incidence rate of 1-2% in the general population. Most patients are unaware of the disease until the onset of infective endocarditis, which is a life-threatening complication that may affect a heart valve or other cardiac structures at the site of endothelial damage. A 22-year-old man presented to our internal medicine clinic with a complaint of acute onset dyspnea and fatigue. His body temperature was 38^ °^C. A diastolic murmur was detected at the right sternal border. Two-dimensional transthoracic echocardiography revealed severe aortic insufficiency, and two-dimensional transesophageal echocardiography showed that the aortic valve was bicuspid. There was also a flail lesion extending the left ventricular outflow tract, resulting in pathological coaptation and severe aortic insufficiency. The patient was referred to our cardiovascular department for surgery. We herein present this case of a bicuspid aortic valve complicated by infective endocarditis due to the underlying disease of chronic otitis media related to a rare pathogen: Alloiococcus otitidis. The patient underwent a successful aortic valve replacement surgery due to aortic insufficiency following infective endocarditis. He was discharged on the 16^th^ postoperative day in good condition.

## Introduction

The bicuspid aortic valve (BAV) is known to be the most common congenital cardiac malformation, with an approximate incidence rate of 1-2% in the general population.^1^ Most patients are unaware of the disease until the onset of infective endocarditis (IE), which is a life-threatening complication that may affect a heart valve or other cardiac structures at the site of endothelial damage.^2^ The etiology of IE may vary according to its potential for causing bacteremia. Chronic otitis media (COM) is a rare condition that may lead to IE.^3^

We describe a patient with a BAV complicated by IE due to the underlying disease of COM.

## Case Report

A 22-year-old man presented to our internal medicine clinic with the complaint of acute onset dyspnea and fatigue. His body temperature was 38 ^°^C. A diastolic murmur was detected at the right sternal border. The laboratory tests revealed anemia with hematocrit of 32% and hemoglobin of 10.4 g/dl. The inflammatory markers were high with sedimentation of 108 mm/h and serum C-reactive protein of 134 mg/L. The leukocyte count was within the normal range, but the neutrophil count was as high as 7.4 10^3^/microL.

**Figure 1:**
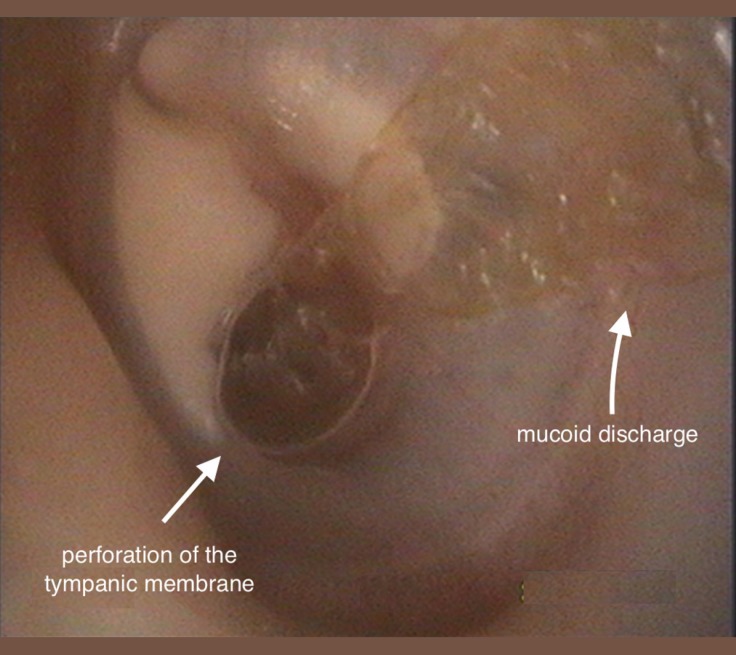
Left ear: presence of chronic otitis media: A) central perforation of the tympanic membrane; B) mucoid discharge; and C) two tympanosclerotic plaques

The patient was referred to the cardiology department for further evaluation of the diastolic murmur and dyspnea. Two-dimensional transthoracic echocardiography revealed severe aortic insufficiency, and two-dimensional transesophageal echocardiography (TEE) showed a BAV as well as a flail lesion extending to the left ventricular outflow tract, resulting in pathological coaptation and severe aortic insufficiency. At first, this unusual lesion was considered to be a vegetation. However, three-dimensional TEE zoom images demonstrated this flailed tissue as a part of the left-right coronary cusp with a defect in the middle part, due to IE. An empiric antibiotic regimen was initiated with Vancomycin and Gentamicin, and a blood sample was taken to adjust the appropriate antibiotic treatment according to the antibiogram. No acute lung edema and acute cardiac failure were diagnosed. For elective surgery, the patient was transferred to our cardiovascular surgery department. After 3 days, the result of the blood culture test was interesting: Alloiococcus otitidis, a Gram-positive coccus as a rare pathogen for IE. Subsequently, he was reevaluated for otitis and the results revealed that there had been COM with intermittent exudative odorless ear discharge for many years since his childhood. He was referred to the otorhinolaryngology department, where a perforated left tympanic membrane with mucoid discharge was detected in the patient (Figure1) and an additional antibiotic drop treatment of local ciprofloxacin was prescribed.

**Figure 2:**
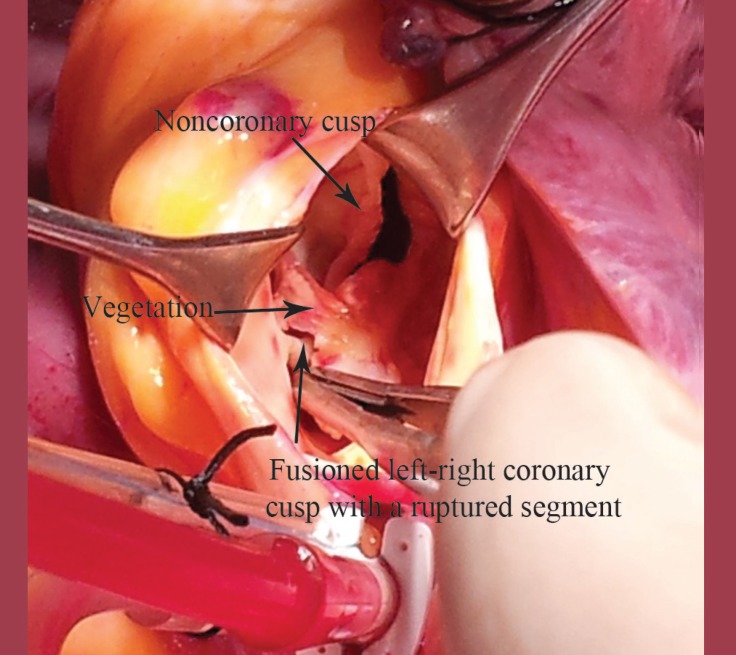
View of the bicuspid aortic valve with a defective left-right coronary cusp

After one week, as the inflammatory markers had decreased, the patient underwent an aortic valve replacement under antibiotic treatment suppression. During the operation, after aortotomy, the aortic valve was observed to have a bicuspid structure with a normal noncoronary leaflet. İn addition, there was a degenerative and fusioned left-right coronary leaflet with a cleft in the middle part (Figure 2). The operation was performed routinely. The resected valve specimens were sent in sterile containers to the microbiology laboratory for Gram staining and culturing and to the histopathology laboratory for histopathological examination and routine processing. The pathology report of the resected aortic valve revealed characteristic acute inflammation signs, polymorphonuclear leucocytes, Gram-positive coccus, and leaflet disruption and thickening. The microbiology culture, however, was negative. 

The patient received the same antibiotic protocol for another 2 weeks and was discharged on the 16^th^ postoperative day without any complications.

## Discussion

The profile of IE has changed over the recent decades in tandem with the increasing survival rates of patients with congenital heart disease. While aortic stenosis itself has a prevalence of 72% in adults with a BAV, primary aortic regurgitation without IE is uncommon in patients with a BAV, as was the case in our patient.^1^ The usual prevalence rate of the IE of BAVs is 10% to 16%. Staphylococci and Viridans streptococci are responsible for most IE cases, accounting for nearly three-quarters of the cases affecting BAVs.^4^ During the last decade, despite all the controversy over whether Alloiococcus otitidis is really a pathogen or a normal bacterial flora,^5^ some investigators have suggested that Alloiococcus otitidis is a pathogen allied to otitis media with effusion.^6^ Apart from these views, our literature review yielded no other reports on Alloiococcus otitidis associated with IE. Most of these infectious disease manifestations of the heart structures have a good prognosis only if diagnosed early and managed appropriately. Our patient was unlucky in that respect. He had two potential risk factors to develop IE: a structural anomaly (BAV) and COM persisting from his childhood, leading to intermittent bacteremia. Unfortunately, he was also unaware of both of these underlying diseases. For a patient with a BAV, COM may be a severe infection source, leading to such catastrophic complications as aortic valve replacement. Combined analysis of clinical, echocardiographic and blood culture results are so vital for the early diagnosis of IE. 

## Conclusion

We suggest that more radical treatment protocols be devised to treat COM for patients prone to IE. We believe that our report on a catastrophic complication of COM, as a rare clinical situation, will contribute to the literature.

## Notes:

***This paper should be cited as: ***Guler A, Sahin MA, Gurkan Yesil F, Yildizoglu U, Demirkol S, Arslan M. Chronic Otitis Media Resulting in Aortic Valve Replacement: A Case Report. J Teh Univ Heart Ctr 2015;10(2):98-100.
